# Probable Hydrochlorothiazide-Induced Myopericarditis: First Case Reported

**DOI:** 10.1155/2015/319086

**Published:** 2015-03-15

**Authors:** Toufik Mahfood Haddad, Muhammad Sarfraz Nawaz, Ahmed S. Abuzaid, Smrithy Upadhyay, Pallavi Bellamkonda, Aryan N. Mooss

**Affiliations:** ^1^Department of Internal Medicine, CHI Health Creighton University Medical Center, Creighton University School of Medicine, 601 North 30th Street, No. 5850, Omaha, NE 68131, USA; ^2^Cardiovascular Medicine Department, CHI Health Creighton University Medical Center, Creighton University School of Medicine, Omaha, NE, USA

## Abstract

Hydrochlorothiazide has never been reported as a reason for myopericarditis. An African American female, with past history of hypertension, coronary artery disease, and sulfa allergy, presented with indolent onset and retrosternal chest pain which was positional, pleuritic, and unresponsive to sublingual nitroglycerin. Her medications included hydrochlorothiazide (HCTZ) which was started three months ago for uncontrolled hypertension. Significant laboratory parameters included erythrocyte sedimentation rate (ESR) of 47 mm/hr and peak troponin of 0.26 ng/mL. Transthoracic echocardiogram (TTE) revealed preserved ejection fraction with no segmental wall motion abnormalities; however, it showed moderate pericardial effusion without tamponade physiology. We hypothesize that this myopericarditis could be due to HCTZ allergic reaction after all other common etiologies have been ruled out. There is a scarcity of the literature regarding HCTZ as an etiology for pericardial disease, with only one case reported as presumed hydrochlorothiazide-induced pericardial effusion. Management involves discontinuation of HCTZ and starting anti-inflammatory therapy.

## 1. Introduction

HCTZ-induced myopericarditis has never been reported in the literature. Despite the low incidence of hypersensitivity reaction to HCTZ, practitioners should have a high clinical suspicion for the development of HCTZ-induced pericardial inflammation given the wide use of HCTZ as an antihypertensive medication and given the high prevalence of sulfa allergy in general population which reaches up to 3–6% [1]. Although sulfonamide antibiotics cross-reactivity with sulfonamide nonantibiotics has rarely been reported [2], concerns should be raised for possible HCTZ-induced pericardial inflammation in patients with sulfa allergy.

## 2. Case Report

**Figure 1:**
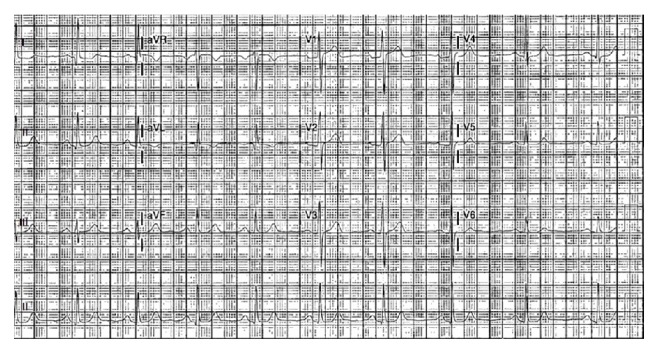
ECG at admission shows left ventricular hypertrophy pattern with unspecific ST-T wave changes.

**Figure 2:**
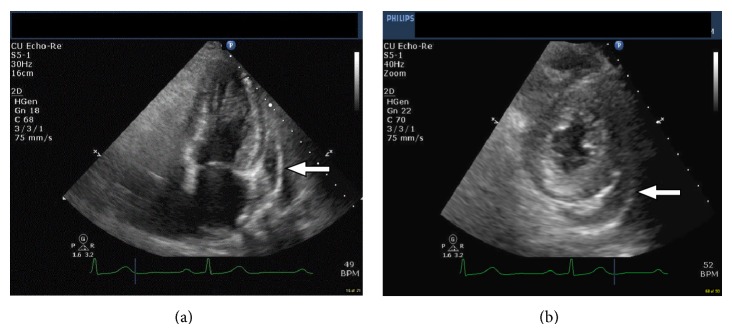
(a) TTE apical long axis view that shows moderate pericardial effusion. (b) TTE midlevel short axis view that shows moderate pericardial effusion.

A 71-year-old African American female presented with indolent onset, positional, pleuritic, and retrosternal chest pain of thirty-minute duration. The pain radiated to the interscapular area, was associated with nausea, and did not respond to sublingual nitroglycerin. She denied any dyspnea on exertion, palpitation, orthopnea, or paroxysmal nocturnal dyspnea. Her past medical history included coronary artery disease with drug-eluting stent placement four years ago, hypertension with hypertensive heart disease, and hyperlipidemia. Patient reported a history of sulfa allergy manifested as rash reaction to sulfamethoxazole-trimethoprim in the past. Her medications included HCTZ, aspirin, metoprolol tartrate, quinapril, and simvastatin. HCTZ was started three months ago for uncontrolled hypertension. Vital signs showed a blood pressure of 164/65 mmHg and heart rate of 65 beats per minute. Cardiac examination revealed muffled heart sounds without any distended neck veins, murmur, extra heart sounds, or pericardial rub. The rest of the physical examination was unremarkable. Significant laboratory parameters included basic metabolic panel, complete blood count, and differential count being within normal range. Troponin I (Tn I) was mildly elevated and peaked at 0.26 ng/mL, with normal creatine kinase (CK) and CK-MB. ESR was slightly elevated at 47 mm/hr with C-reactive protein and thyroid-stimulating hormone being within normal range. Other negative work-ups included rheumatoid factor, antinuclear antibodies, tuberculosis skin test, human immunodeficiency virus (HIV) antibodies, hepatitis virus panel, and urine drug screen. Chest X-ray revealed enlarged cardiac silhouette without any pulmonary vascular congestion. An electrocardiogram (EKG) revealed normal sinus rhythm, nonspecific ST-T waves changes, and left ventricular hypertrophy pattern (Figure 1). Transthoracic echocardiogram (TTE) revealed normal left ventricular systolic function without segmental wall motion abnormalities and a moderate pericardial effusion without tamponade physiology (Figures 2(a) and 2(b)). Because of her symptoms of chest pain, elevated troponin, and significant history of coronary artery disease, Technetium-99 Single-Photon Emission Computed Tomography (SPECT) myocardial perfusion imaging (MPI) at rest and with stress was done which showed small fixed perfusion defect in the apex likely related to prior myocardial infarction, without any reversible perfusion defect, which made acute coronary syndrome less likely to be the reason of her symptoms supported by the lack of ECG ischemic changes, left ventricular wall motion abnormalities, or any decompensated ejection fraction.

Based on chest pain typical for pericardial disease, slightly elevated cardiac biomarkers, and moderate pericardial effusion, the patient was diagnosed with acute myopericarditis, with the potential etiology being HCTZ, given her history of known sulfa allergy and recent initiation of this medication. The plausibility of pericardial effusion secondary to an immunologic-hypersensitivity reaction was considered. She was treated with aspirin for symptomatic management and was advised to avoid HCTZ in the future. Her pericardial effusion resolved on one-month follow-up.

## 3. Discussion

Myopericarditis is a term used to prescribe the majority of patients with primarily pericarditis with minor myocardial involvement [3]. Pericarditis is diagnosed by two of the following four points including typical chest pain, pericardial fraction rub, new or worsening pericardial effusion, and suggestive electrocardiographic changes (diffuse ST segment elevation or PR depression) [3, 4]. Although, elevated C-reactive protein (CRP) is considered a confirmatory factor required for diagnosis of acute pericarditis [5]; it is elevated in only 78% cases at presentation [6]. Pericarditis and myocarditis usually share etiologies including infections, trauma, radiation, neoplastic, postmyocardial infarction, drugs, autoimmune diseases, and metabolic such as hypothyroidism; thus, a spectrum of myopericardial inflammatory syndromes, ranging from pure pericarditis to myopericarditis (predominant pericarditis), perimyocarditis (predominant myocarditis), and pure myocarditis, has been described [7].

Proposed diagnostic criteria for myopericarditis include evidence of pericarditis plus elevation of markers of myocardial inflammation, that is, troponin, or myocardial inflammatory involvement assessed by an imaging method, that is, cardiac magnetic resonance, but without wall motion abnormalities and reduced LV function, while in the case of wall motion abnormalities and/or reduced LV function, the term “perimyocarditis” will be used [4].

Due to benign prognosis of pericarditis, further work-up is not warranted in immunocompetent patients unless there is an apparent correlation or medical condition [8]. In the presence of features suggesting associated disease, the likelihood of this being the cause for the pericardial syndrome is very high. Therefore, associated diseases should be investigated accordingly [9]; however, most of the cases remain idiopathic. Pericardiocentesis is necessary in the presence of pericardial tamponade or large effusion refractory to conservative management and with severe symptoms, or when bacterial or neoplastic pericarditis is suspected [3, 4, 8]. Similarly myocardial biopsy is only indicated when there is fulminant myocarditis manifesting as symptomatic severe left ventricular systolic dysfunction after the exclusion of CAD as an etiology [10].

Medications are one of the rare causes of pericardial inflammation with different mechanisms including drug-induced lupus, idiosyncrasy or hypersensitivity, serum sickness, foreign substance reactions, and immunopathy [11]. Sulfa drugs including thiazides, mesalazine, amiodarone, and bromocriptine are categorized under idiosyncratic reaction [11], which is characterized by being unpredictable, unrelated to dose, and resolving only on discontinuation of the drug. HCTZ-induced myopericarditis has not been reported before in the literature with only one case reported as presumed hydrochlorothiazide-associated, immunologic-hypersensitivity-induced pericardial effusion [12]. Thiazides are considered one of the culprits since they contain a sulfa group, which can activate mast cells by immunoglobulin E mediated immune reaction, manifesting as allergic-like reaction to sulfonamide-containing nonantibiotics in patients with known allergies to sulfonamide-containing antibiotics [2].

In general, myopericarditis requires admission for monitoring and therapy. Anti-inflammatory drugs should be given only for symptoms management considering the fact they may enhance the myocardial inflammation and necrosis and may also increase mortality [7].

Lower doses of anti-inflammatory drugs are usually prescribed mainly to control symptoms for one to two weeks rather than reaching full anti-inflammatory effects such as in simple pericarditis [7]. A beta-blocker may be considered, with the addition of an ACE-inhibitor in cases with regional or global LV dysfunction [13]. Exercise restriction is recommended for at least 4 weeks, as well as regular echocardiographic monitoring of ventricular function, especially in patients with left ventricular dysfunction [7]. The management of pericardial disease in case of drug reaction is based on the discontinuation of the causative agent and symptomatic management [11].

## 4. Conclusions

We hypothesize that this myopericarditis was secondary to HCTZ with patient's history of allergy to sulfonamide antibiotics. This should raise the drug reaction as a differential diagnosis for any case of pericardial disease with unknown etiology. Hence, it is very important to review all medications carefully to address the potential culprit ones.
